# Mortality and Health Outcomes in HIV-Infected and HIV-Uninfected Mothers at 18–20 Months Postpartum in Zomba District, Malawi

**DOI:** 10.1371/journal.pone.0044396

**Published:** 2012-09-04

**Authors:** Megan Landes, Monique van Lettow, Richard Bedell, Isabell Mayuni, Adrienne K. Chan, Lyson Tenthani, Erik Schouten

**Affiliations:** 1 Dignitas International, Zomba, Malawi; 2 Department of Family and Community Medicine, University of Toronto, Toronto, Ontario, Canada; 3 Dalla Lana School of Public Health, University of Toronto, Toronto, Ontario, Canada; 4 St. Michael’s Hospital, University of Toronto, Toronto, Ontario, Canada; 5 Department of HIV and AIDS, Ministry of Health, Lilongwe, Malawi; 6 Management Sciences for Health, Lilongwe, Malawi; Indiana University, United States of America

## Abstract

**Background:**

Maternal morbidity and mortality among HIV-infected women is a global concern. This study compared mortality and health outcomes of HIV-infected and HIV-uninfected mothers at 18–20 months postpartum within routine prevention of mother-to-child transmission of HIV (PMTCT) services in a rural district in Malawi.

**Methods:**

A retrospective cohort study of mother-child dyads at 18–20 months postpartum in Zomba District. Data on socio-demographic characteristics, service uptake, maternal health outcomes and biometric parameters were collected.

**Results:**

173 HIV-infected and 214 HIV-uninfected mothers were included. HIV-specific cohort mortality at 18–20 months postpartum was 42.4 deaths/1000 person-years; no deaths occurred among HIV-uninfected women. Median time to death was 11 months post-partum (range 3–19). Women ranked their health on a comparative qualitative scale; HIV-infected women perceived their health to be poorer than did HIV-uninfected women (RR 2.4; 95% CI 1.6–3.7). Perceived maternal health status was well correlated with an objective measure of functional status (Karnofsky scale; p<0.001). HIV-infected women were more likely to report minor (RR 3.8; 95% CI 2.3–6.4) and major (RR 6.2; 95% CI 2.2–17.7) signs or symptoms of disease. In multivariable analysis, HIV-infected women remained twice as likely to report poorer health [adjusted OR (aOR) 2.3; 95% CI 1.4–3.6], as did women with low BMI (aOR 2.1; 95% CI 1.1–4.0) and scoring lowest on the welfare scale (aOR 2.0; 95% CI 1.1–3.8).

**Conclusions:**

HIV-infected women show increased mortality and morbidity at 18–20 months postpartum. In our rural Malawian operational setting, where there is documented under-application of ART and poor adherence to PMTCT services, these results support attention to optimizing maternal participation in PMTCT programs.

## Introduction

The reduction of maternal mortality remains one of the greatest challenges in global health [1]. In particular, countries in sub-Saharan Africa (SSA) continue to report alarmingly high maternal mortality rates [2] and this has been, in part, attributed to the HIV epidemic. HIV-infection has been shown to increase a woman’s risk for obstetrical complications, as well as for illness during the postpartum period [2], [3], [4]. During the 1990s, a 10-fold increase in the prevalence of HIV-infection among women was reported in Malawi, along with a concurrent doubling of the overall pregnancy-related mortality risk [5].

During the past decade, there has been considerable progress in SSA to scale up prevention of mother-to-child transmission of HIV (PMTCT) services aimed to identify HIV-infection in pregnancy for the prevention of vertical transmission, but also to refer mothers for appropriate antiretroviral treatment (ART) services when available and indicated.[6]–[8] Despite these efforts to increase access to ART for women, several studies still show considerable increased risk of mortality amongst HIV-infected women in the postpartum period as well as an elevated incidence of morbidity [9]–[13].

From 2003 to 2010, the primary prophylaxis regimen within the Malawian PMTCT strategy was single dose nevirapine (sd-NVP). At the time of study (2008–2009), PMTCT services included: routine opt-out HIV testing and counselling (HTC) for women presenting to antenatal clinics (ANC) and maternity wards, WHO clinical staging and CD4 count if indicated (for WHO clinical stage I or II) and available, initiation of highly active antiretroviral treatment (ART) for women in WHO clinical stages III or IV or in stages I and II if CD4 count <250 cells/mm^3^, single dose nevirapine (sd-NVP) for women not initiated on ART and for all HIV-exposed infants, and follow-up of exposed infants up to 18 months.

We previously report within an operational research study evaluating the uptake of PMTCT services in Zomba District, the suboptimal coverage of PMTCT services including only 66% percent of women taking sd-NVP at the time of delivery and 28% of exposed infants being tested for HIV [14]. As part of this previously reported study, we additionally collected data on maternal deaths and health outcomes at 18–20 months postpartum for which we report on in this current study. This information describing maternal outcomes within this rural district of Malawi under routine program conditions has value as a baseline reference for future program evaluation since, as of July 2011, Malawi is the first country to begin implementing a universal “test and treat” strategy known as ‘Option B Plus’. This national program will start all HIV-infected pregnant women on lifelong ART regardless of WHO CD4 count with the overall goal to streamline ART application and improve outcomes of HIV- infected pregnant women and their infants in the continuum of PMTCT services [15].

## Methods

### Study Setting, Study Design and Data Collection

Data were collected as part of a retrospective cohort study in Zomba District (population 670,000; 80% rural [16]), in south-eastern Malawi where antenatal surveillance data estimates an HIV-prevalence of 12–24% [17]. The methods used in this study have been described in detail elsewhere [14]. Twenty of 22 sites in the district where PMTCT services had been available for at least 18 months were included in this study. Two large hospitals serving as referral centres for the South-East Zone of Malawi (Zomba Central Hospital and St. Luke’s Mission Hospital), where approximately one quarter of all HIV-infected mothers in the district give birth, were excluded as women who gave birth at these sites may not live within the Zomba district and therefore would be difficult to trace. Antenatal registers in these 20 rural public health facilities were reviewed to include all women with an estimated delivery date (EDD) between March 1st and May 31st 2008. Additionally we reviewed all delivery and postpartum registers during the specified period in an attempt to capture all HIV-infected pregnant women in the district with a documented delivery between March 1^st^ and May 31^st^, 2008. For every HIV-infected mother, the next registered HIV-uninfected mother was identified as a control.

Lay community health workers employed by the Malawi Ministry of Health (MoH), known as Health Surveillance Assistants (HSAs), who were already working in the catchment areas of the individual rural health facilities helped trace the identified women for the purpose of this study. The HSA’s asked the identified mother-child pairs to come to the health centre for interviews and, in cases where the mother had died, the child’s primary caregiver was asked to attend for interview. If the mother or the primary caregiver did not come for interview after having agreed to come, then the interviewer, accompanied by the HSA, visited the participant at home for the interview. In cases where study participants were reported to have moved or died, village headmen were consulted to confirm this information and village registers reviewed for verification.

Data collection was conducted by 6 trained female interviewers using semi-structured interviews from a standardized questionnaire. Information concerning prior HIV-testing and ART was verified through personal health passports.

Specifically for this analysis, socio-demographic information was collected, including socio-economic indicators (i.e. level of education, housing type, water source, household size, source of income and means of transport). Socio-economic indicators were selected using the household poverty assessment model for Malawi as described by Payongayong et al [18]. The selected variables were ranked from poorest to wealthiest; a rank sum of all the ranked variables was made and categorized in percentiles, with 1 as the poorest and 4 the wealthiest. Data was collected on the health status of all women surviving to the time of study (i.e.18–20 months postpartum); no information was available on health status for those women who had died prior to the time of study. Women were asked in the local language (Chichewa) to report their perceived health on a comparative qualitative scale as “excellent”, “good”, “fair” or “poor”. “Poor perceived health” was categorized as all rankings other than “excellent”. Interviewers then ranked maternal functional health status on the Karnofsky scale based on a series of questions. The Karnofsky scale ranks function based on activity level and symptoms of disease and has been used frequently in the context of measuring outcomes in HIV-infected cohorts in Africa [19]–[21]. Weight and height were obtained at the time of interview, and low body mass index was considered as less than 18.5 kg/m^2^.

Mothers and/or children with negative or unknown HIV status were offered point of care HIV rapid testing at the time of study. All testing was performed by trained counsellors as per the Malawi MoH National Guidelines and all persons with positive HIV tests were referred for assessment of eligibility for ART.

### Statistical Analysis

Data and statistical analysis were conducted using STATA 11.0 (StataCorp LP, College Station, Texas, USA). Baseline characteristics were summarized using means for continuous variables and proportions for categorical variables and compared using Student *t*-tests and χ2 tests, respectively. Relative risks (RR) were used as a measure of associations between HIV status and perceived and functional health status. Risk factors for reporting of worse health status were determined through multivariate logistic regression, described as adjusted odds ratios (aOR).

### Ethics

This study received ethical approval from the National Health Science Research Committee in Malawi. Written informed consent was obtained from all participants involved in the study.

## Results

### Maternal Characteristics

360 HIV-infected mothers and 360 HIV-uninfected mothers were identified through all available registers. 173 HIV-infected and 214 HIV-uninfected mothers were found and included in the study. Out of the 720 mother-child pairs traced, 61 had moved out of the area, 269 were not found, and 3 were not willing to participate. No significant difference was found between HIV-infected and HIV-uninfected women in the proportion having moved out of the area or not found (p = 0.09).

*Table 1* shows descriptive characteristics by HIV status for all women. In addition to being more likely to be older and less educated than HIV-uninfected women [14], HIV-infected women were also more likely to be single, divorced/separated or widowed (p<0.001), to be the head of their household (22.5% vs. 8.9%; p<0.001) and to be poor (27.8% vs. 22.4%; p = 0.004).

**Table 1:** Maternal characteristics by HIV status at 18–20 months postpartum.

		HIV-infected (N = 173)	HIV-uninfected (N = 214)	p-value
**Mean Age (yrs)**		29.1 (SD 5.8)	25.3 (SD 5.6)	<0.001
**Marital Status**	Married	125 (72.3)	183 (85.5)	<0.001
	Single	12 (6.9)	7 (3.3)	
	Divorced/Separated	24 (13.9)	24 (11.2)	
	Widowed	7 (4.1)	0 (0)	
	Unknown	5 (2.9)	0 (0)	
**Median Parity**		4 (IQR 1–7)	2 (IQR 0–5)	<0.001
**Household Head**	Husband	120 (69.4)	182 (85.0)	<0.0001
	Self	39 (22.5)	19 (8.9)	
	Other	9 (5.2)	13 (6.1)	
	Unknown	5 (2.9)	0 (0)	
**Welfare Scale** *	Poorest	48 (27.8)	48 (22.4)	0.004
	2	48 (27.8)	57 (26.6)	
	3	43 (24.9)	44 (20.6)	
	Wealthiest	29 (16.8)	65 (30.4)	
	Unknown	5 (2.9)	0 (0)	
**ART at time of study**	On ART	45 (26.0)		
	Not on ART (CD4>350)	56 (32.3)		
	Not on ART (Never Staged and/orno CD4 count)	72 (41.6)		

* A composite variable derived from multiple responses (e.g. level of education, housing type, water source, household size, source of income and means of transport).

We previously report the use of highly active ART during pregnancy and delivery of the 173 HIV-infected mothers: 9 women were on ART before attending the ANC clinic, 100 (65%) of the mothers not already on ART at ANC reported they were referred to and attended pre-ART services at least once, and 8 (5%) mothers initiated ART during pregnancy. Overall, 17 mothers were on highly active ART during pregnancy and delivery [14].

*Table 1* also describes ART use for all HIV-infected women at 18–20 months (at the time of study). 45 (26%) HIV-infected mothers were on ART and of the 128 HIV-infected mothers not on ART, 56 (44%) reported not having started based on high CD4 and WHO Staging of I or II. The remaining 72 HIV-infected mothers (56%) not on ART were either never staged or had a WHO Stage of I or II and had an unknown CD4 count.

### Maternal Deaths

We report no deaths among HIV-uninfected women at 18–20 months postpartum, and an HIV-specific cohort mortality rate of 42.4 deaths/1000 person years. The median time to death was 11 months postpartum (range: 3 to 19) and the median age for women at time of death was 25 years (range: 18 to 50; missing one data point).

Neither maternal age nor home birth was associated with maternal death. Further demographic information was available for 6 of the 11 mothers who died. In comparison to surviving HIV-infected women, mothers who died were more likely to be widowed (50% vs. 2.4%, p<0.001), less likely to have a husband as the household head (33.3% vs. 72.8%; p<0.001) and more likely to have another relative other than themselves acting as head of household (66.7 vs. 3.0%; p<0.001).

### Maternal Health Outcomes at 18–20 Months

#### Reproductive health

HIV-infected women had significantly higher parity than HIV-uninfected mothers; when controlled for age, HIV-infected women were still likely to have higher parity [14]. Within the total cohort, 64 (16.5%) women had a previous stillbirth and 4 women (1.0%) had a stillbirth in this index pregnancy. There was no association between previous stillbirth or current stillbirth and HIV-infection (p = 0.43 and p = 0.71). Additionally, in this pregnancy, 36 (9.3%) women had a preterm delivery, which was not associated with HIV-infection (p = 0.08).

At the time of data collection (i.e. 18–20 months postpartum), 40 (10.6%) women were pregnant again. Women whose child had died in the index pregnancy (i.e. mother-child pair included for cohort study) were more likely to be pregnant again at 18–20 months postpartum than women with a surviving child [32 (22.9%) vs. 9 (9.4%); p = 0.01].

#### Health status of mothers

When asked to rate their own health status on a comparative scale, 51.3% of all women reported being in “excellent health”, 31.6% as “good”, 15.7% as “fair” and only 1.3% reported “poor”. Perceived health status on this scale was strongly correlated with the concurrent measure of functional health on the Karnofsky scale (p<0.001).

*Table 2* shows health outcomes of mothers at 18–20 months postpartum by HIV status. In the total cohort, 54 (14%) women had a low BMI (<18.5), and this was not significantly associated with HIV-infection or with available sociodemographic variables (not in Table). Being pregnant at 18–20 months postpartum pregnancy was not associated with BMI. Among women with HIV-infection, 23 of 141 (16%) women with known BMI’s had a low BMI (consistent with advanced clinical disease) and only 9 of those were on ART (39%).

**Table 2:** Health status of surviving mothers at 18–20 months postpartum by HIV status.

		HIV-infected(N = 173)	HIV-uninfected (N = 214)	RR* (95%CI)	p-value
**Currently pregnant**	No	139 (85.8)	194 (90.7)		0.409
	Yes	22 (13.6)	19 (8.9)		
	Unknown	1 (0.6)	1(0.5)		
**Mother’s BMI**	Underweight (<18.5)	23 (13.3)	31 (14.5)		0.094
	Normal (18.5–24.9)	120 (69.4)	162 (75.7)		
	Overweight (>25)	21 (12.1)	18 (8.4)		
	Unknown	9 (5.2)	3 (1.4)		
**Perceived health status**	Excellent	63 (38.9)	130 (60.8)	1	0.001
	Good	61 (37.7)	58 (27.1)	2.2 (1.4–3.5)	
	Fair	33 (20.4)	26 (12.2)	2.6 (1.4–4.8)	
	Poor	5 (3.1)	0	–	
**Functional health status**	Normal activity/work	86 (53.1)	179 (83.6)	1	0.001
	Normal activity/work; minor signsand symptoms of disease	53 (32.7)	29 (13.6)	3.8 (2.3–6.4)	
	Normal activity/work; major signsand symptoms of disease	15 (9.3)	5 (2.3)	6.2 (2.2–17.7)	
	Unable to work; can performself-care	2 (1.2)	1 (0.5)	4.2 (0.4–46.5)	
	Unable to work; needs occasionalassistance for self-care	2 (1.2)	0	.	
	Unable to care for self; needsconsiderable assistance	4 (2.5)	0	.	
	Unable to care for self; severelydisabled/total care	0	0	.	

* Unadjusted for other variables.

On the perceived health scale, HIV-infected women were twice as likely to report their health as only “good” (RR 2.2; 95% CI 1.4–3.5) or “fair” (RR 2.6; 95% CI 1.4–4.8) compared with HIV-uninfected women. Risk factors for worse perceived health are shown in *Table 3*. In multivariable analysis, worse perceived health was significantly associated with: HIV-infection (aOR 2.3; 95% CI 1.4–3.6), low BMI (aOR 2.1; 95% CI 1.1–4.0) and being poor (aOR 2.0; 95% CI 1.1–3.8). Among the subset of HIV-infected women, there was no association between health status and their uptake of PMTCT services (acceptance of HTC, taking sd-NVP at birth, baby taking sd-NVP at birth) or their current use of ART.

**Table 3:** Factors associated with poor perceived health status.

		Excellent Health(N = 193)	Less than ExcellentHealth (N = 183)	Unadjusted OR(95% CI)	Adjusted OR*(95% CI)	p-value
**HIV status**	Uninfected	130 (67.4)	84 (45.9)	1.0	1.0	.
	Infected	63 (32.6)	99 (54.1)	2.4 (1.6–3.7)	2.3 (1.4–3.6)	0.000
**BMI**	Low (<18.5)	19 (9.8)	35 (19.1)	2.0 (1.1–3.7)	2.1 (1.1–4.0)	0.018
	Normal (18.5–24.9)	147 (76.2)	133 (72.7)	1	1.0	.
	High (>25.0)	26 (13.5)	13 (7.1)	0.6 (0.3–1.1)	0.5 (0.2–1.0)	0.044
**Welfare status**	Poorest	42 (21.8)	51 (27.8)	2.4 (1.3–4.2)	2.0 (1.1–3.8)	0.021
	2	47 (24.4)	56 (30.6)	2.3 (1.3–4.1)	1.8 (1.0–3.2)	0.051
	3	42 (21.8)	44 (24.0)	2.0 (1.1–3.7)	1.7 (0.9–3.1)	0.139
	Wealthiest	62 (32.1)	32 (17.5)	1.0	1.0	.
**Total no. of dependents**	0–2	94 (48.7)	69 (37.7)	1	1.0	.
	3–5	87 (45)	92 (50.3)	1.4 (0.9–2.2)	1.5 (0.9–2.5)	0.219
	6 or >	12 (6)	22 (12)	2.5 (1.2–5.4)	2.6 (1.0–6.7)	0.047

* Adjusted for all other variables in the table along with age and parity.

On the functional health scale, HIV-infected women were four times more likely to have minor signs and symptoms of disease (RR 3.8; 95% CI 2.3–6.4) and six times more likely to report major signs and symptoms of disease than HIV-uninfected women (RR. 6.2; 95% CI 2.2–17.7). Additionally, amongst those HIV-infected women reporting minor or major symptoms of disease, current ART was markedly under-applied: only 20% of the HIV-infected women reporting major signs and symptoms of disease were on ART at the time of study (3/14), and only 50% of the women reporting needing some assistance for daily living (1/2) or considerable assistance were on ART (2/4). Among these 14 women reporting symptoms of disease and who were not on ART, the reasons given for not being on ART were: high CD4 count or “no need to start ART yet” (4/14), referred for ART services but didn’t go (3/14) or were never told nor referred (8/14).

## Discussion

In this study we document an alarming mortality rate at 18–20 months postpartum of 42.4 deaths/1000 person years amongst HIV-infected women. Several other studies have demonstrated similar rates amongst HIV-infected women at 2 years postpartum, with a recent South African study reporting 55.7 deaths/1000 person years and a Zimbabwean study reporting 36.5 deaths/1000 person years [10], [22].

This high mortality we report in HIV-infected women also indicates a markedly elevated relative risk of mortality in comparison to that of HIV-uninfected women (there were no deaths among HIV-uninfected women). It is reasonable to conclude that this observed mortality is likely to be HIV-related and is similar in magnitude to that of an observational cohort in two Malawian urban centres which found at 12 months postpartum a 2.0% (25/1222) mortality among HIV-infected women and no deaths among the HIV-uninfected women [9]. An increased risk of mortality in the postpartum period amongst HIV-infected women in comparison to HIV-uninfected women has also been documented in SSA, although the magnitude of this risk varies from very large as reported in Zimbabwe (RR 54.1) [10] to smaller relative risks in Uganda (RR 5) and Congo (RR 4) [23], [24]. These results may reflect differing study population characteristics (including differences in rural vs. urban), as well as variable access to obstetrical or HIV-related services, specifically diagnosis and treatment for TB or other opportunistic infections, and access to ART.

In this study, HIV-infected women also had significantly greater morbidity than HIV-uninfected women at 18–20 months postpartum. In Zambia, HIV-infected women were more likely to report physical morbidity during the postpartum period [13] and in South Africa, high levels of serious morbidity at two years postpartum amongst HIV-infected women, including a high incidence of pulmonary TB, have also been reported [22]. Other studies have demonstrated significant rates of malaria, diarrhea and pneumonia amongst HIV-infected women in the postpartum period [9], [25].

In our study, reporting of poor maternal health status was also associated with low socioeconomic status. One study lower socioeconomic indicators to be associated with a shorter time to first serious HIV-related morbidity event and this may be mediated by lower levels of education, income and/or access to health services [22]. Additionally, a cohort from Cape Town has demonstrated an association between lower levels of income and increasing loss to follow-up, which may in turn lead to worse health outcomes in this population [26].

Finally, we show a significant association between low BMI (<18.5) and poor health status within the entire cohort and within the subset of HIV-infected women. Low BMI and/or severe wasting occurs in approximately one third of all cases of advanced HIV and is a strong risk factor for poor health outcomes in the non-pregnant population [27]–[30]. Low BMI in this cohort may also represent malnutrition, in particular micronutrient malnutrition, which has been shown to predict worse health outcomes [28]. Several studies have shown a link between low BMI and an increased incidence of TB in HIV-infected patients [29], [31], which could partially explain an unmeasured cause of worse health status in this cohort of women.

We have previously reported low uptake of ART within this cohort in the context of PMTCT services: only 10% were either already on/or initiated on ART before delivery of this index pregnancy and by 18–20 months postpartum only 26% HIV-infected mothers were on ART. Of those not on ART, 44% reported not having started ART based on high CD4 counts but the remaining 56% were either never staged or had an unknown CD4 [14]. Thus, there remains a large proportion of HIV-infected women for whom ART was potentially indicated and under applied. There is increasing evidence of significant loss to follow-up in postpartum women within PMTCT programs in the region [32] which may contribute in this cohort to the lack of staging or CD4 count acquisition, however improved access to ART is warranted based on this study, and enhanced attention to the application of maternal ART [33].

### Limitations

This study was limited to the cohort of women traced, with significant loss to follow-up of both HIV-infected (51%) and uninfected (41%) women. While these proportions were not significantly different (p = 0.09), we cannot comment on the characteristics of these women lost to follow-up. Thus, a systematic bias may exist in sampling and lead to an underestimation of mortality and morbidity, as other studies in the literature suggest both may be high among HIV-infected women lost to follow-up in PMTCT programs, and may also have other unpredictable impacts on the results. Additionally, this loss to follow-up has lead to a selection bias in that we can only report on the health outcomes of women surviving to 18–20 months and not on the entire cohort.

We acknowledge the limitation within this study of lack of data regarding the health status or ART use of those women who had died and the WHO staging and CD4 counts of the HIV-infected cohort in relation to the mortality. We report previously within this study that 41% of women had not been staged or had a CD4 count and we perceive the lack of practical access to staging and CD4 counts as two major implementation gaps limiting access of pregnant women to ART. In turn, we identify underuse of ART in our cohort as a major factor in the maternal mortality we observed [14]. As operational research, we believe that this lack of data on these issues is not a *study shortcoming* so much as it is a *health systems shortcoming* that the study describes, and it is crucially related to the maternal mortality being reported.

Additionally, this study did not include women that were registered only at tertiary referral centres and it is possible that these women had different characteristics and health outcomes than our study population (e.g. urban, wealthier, high risk obstetrical problems).

### Conclusions

In an operational setting of PMTCT program scale up of single-dose NVP in a rural Malawian District, morbidity and mortality remain high, and are correlated with substantial under-application of ART. In our setting, the significantly poorer health status among HIV-infected women postpartum strongly suggests that they may already have had undetected symptoms for HIV disease upon presentation to ANC clinics. Our experience in Zomba District argues for earlier and more comprehensive assessment and institution of indicated ART in the PMTCT cascade with all of its attendant benefits in similar settings.

In Malawi, some of this measured maternal morbidity and mortality may be mitigated by the current application of Option B Plus [15], a novel national PMTCT policy to implement a universal test and treat strategy. Under this Option B Plus policy, which began roll-out in July 2011, all pregnant women regardless of CD4 count are being started on lifelong ART. Several issues identified at the national level are potential threats to the successful implementation and scale up of Option B including the potential for suboptimal uptake of ART by pregnant women due to stigma and fear of reprisal, as well as low adherence to ART and lack of support for long term retention in this relatively asymptomatic patient population [15]. This study provides baseline comparative data for operational research regarding the impact of this new policy on maternal and infant mortality.
